# The Heat about Cultured Meat in Poland: A Cross-Sectional Acceptance Study

**DOI:** 10.3390/nu15214649

**Published:** 2023-11-02

**Authors:** Dominika Sikora, Piotr Rzymski

**Affiliations:** 1 Department of Environmental Medicine, Poznan University of Medical Sciences, 60-806 Poznan, Poland; rzymskipiotr@ump.edu.pl; 2 Doctoral School, Poznan University of Medical Sciences, Fredry St. 10, 61-701 Poznan, Poland

**Keywords:** in vitro meat, consumer’s acceptance, cultivated meat, consumer’s openness, consumer’s attitude, cell-based meat, lab-grown meat

## Abstract

Cultured meat, produced by culturing animal cells in vitro, is gaining increasing interest. The first products obtained using this technology were authorized for human consumption in Singapore and the United States, and more are likely to follow in other parts of the world. Therefore, it is important to assess the attitudes toward such meat in various populations and understand the grounds for its acceptance and rejection. The present cross-sectional online study of adult Poles (*n* = 1553) aimed to evaluate knowledge of cultured meat, the main reasons and fears associated with its production and consumption, and willingness to buy it and factors influencing such willingness. Most respondents (63%) were familiar with the concept of cultured meat, and 54% declared to purchase it when available. However, concerns over safety were expressed by individuals accepting (39%) and rejecting (49%) such meat. The main motivations for choosing it included limiting animal suffering (76%) and environmental impacts of meat consumption (67%), although over half of responders willing to buy these products were driven by curiosity (58%). Multiple logistic regression revealed that odds (OR; 95%CI) for accepting cultured meat were significantly increased for adults aged 18–40 (1.8; 1.2–2.7); women (1.8; 1.2–2.7); meat eaters (8.7; 5.6–13.6); individuals convinced that animal farming adversely affects the climate (7.6; 3.1–18.3), surface waters (3.1; 1.2–8.1), and air quality (3.0; 1.2–7.6); those familiar with cultured meat concept (4.2, 2.2–8.4); and those revealing high openness to experience (1.7; 1.2–2.4). The results highlight that the Polish population may be moderately ready to accept cultured meat and identify the groups resistant to accepting it. Well-designed and transparent promotion of these products is required to increase the general public’s understanding of the potential benefits and challenges of cultured meat technology.

## 1. Introduction

According to prediction, the demand for animal proteins will continue to increase in various world regions (OECD and FAO, 2021). However, industrial animal agriculture, which is a primary model of meat production, comes with numerous adverse impacts, limitations, and challenges. First, this sector is highly vulnerable to viral diseases that can cause substantial economic loss and disturbance in the food market, e.g., highly pathogenic avian influenza or African swine fever [1,2]. Second, the production of animal products is also related to the potential risks of transmission of various human pathogens, including viruses, bacteria, microsporidia, parasitic worms, protozoa, and prions [3,4]. Third, the extensive use of antibiotics in livestock (that exceeds by three-fold the volume consumed by humans) promotes resistance, which is an increasing threat leading to increased morbidity and mortality due to bacterial infections [5,6]. Fourth, meat production is associated with significant environmental impacts, including soil, water, and air pollution, greenhouse emissions, land use change, water consumption, and biodiversity loss [7,8,9,10]. Fifth, protein feed conversion of meat production is low, approximately 20% for poultry, 8.5% for pork, and 4% for beef [11]. Last but not least, animal husbandry is a subject of ethical concern, especially considering its scale. In 2021 alone, nearly 74 billion chickens, 1.4 billion pigs, 617 million sheep, 501 million goats, and 332 million cows were slaughtered for meat production [12].

Although plant-based diets could be seen as a solution to most issues associated with meat production while providing some health benefits, a vast majority of consumers are unwilling to change their dietary patterns radically [13]. Similarly, insect-based proteins may offer some advantages, including lessening environmental impacts and better feed protein conversion, but their low acceptance, driven by disgust toward entomophagy, particularly in developed countries, remains a significant obstacle [14]. Cultured meat, defined as meat produced from in vitro cell cultures without slaughtering animals [15], is considered superior to conventional meat, particularly regarding protein feed conversion, environmental impacts, and public health issues, while not requiring the elimination of meat from diet or the introduction of completely novel foods [16,17,18].

Cultured meat technology has received substantial interest from the private sector and selected national stakeholders. In December 2020, the Singapore Food Agency became the first regulator to approve cultured meat products, i.e., cell-cultured meat [19]. In 2023, the U.S. Department of Agriculture also authorized such products following a pre-market safety assessment by the Food and Drug Administration [20,21,22]. As yet, cultured meat is not approved in Europe, although the advisory body, the European Food Safety Authority (EFSA), has ensured its readiness for assessment of the potential application regarding cultured meat [23], while in 2022, companies investing in cultured meat technology launched a coalition committed to developing and supporting cultured meat in the European area [24].

Despite the potential benefits of introducing cultured meat to the market as an alternative to conventional products and the easiness of implementing it into the diet without excluding animal-derived foods, the question arises regarding the public acceptance of such products in various world parts. Lessons from genetically modified foods’ introduction clearly show that the ill-planned promotion of food produced by new technology may lead to misinformation, ultimately resulting in reluctant attitudes, which may have a long-lasting effect [25,26,27,28]. In the case of other alternative proteins, acceptance can be increased by informing on their nutritional properties, comparing them to conventional meat, emphasizing advantages related to sustainability and ethics, and addressing safety concerns expressed by consumers [29,30]. The attitude towards cultured meat may also vary depending on various individual factors and characteristics of particular populations, including the level of openness to novelty [26,31,32]. Poland is characterized by a conservative approach towards science as indicated by a highly negative attitude toward genetically modified organisms [25] or opposition against vaccinations as evidenced by COVID-19 vaccine hesitancy, low influenza vaccine coverage, increasing refusal rate of mandatory children’s vaccinations, and late introduction of national vaccinations against human papillomavirus [33,34,35,36,37]. Therefore, it is likely that Poland may represent a particularly challenging European region for introducing cultured meat, although studies directly assessing it are required.

The present study aimed to evaluate the perception of Polish adults toward cultured meat and understand the role of potential factors influencing its acceptance and rejection, such as demographics, education, dietary patterns, openness to experience, and attitudes toward the impact of animal farming on the environment. Main motivations and concerns over cultured meat were also explored. Understanding the attitudes toward cultured meat prior to its introduction is essential to guide stakeholders and food authorities in shaping communication with consumers and building trust in novel foods and production technologies.

## 2. Materials and Methods

### 2.1. Survey

The study was based on an anonymous, self-designed, structured questionnaire available online between March and July 2023. The questionnaire was promoted through a media release by the Polish Press Agency (the single largest source of news in Poland) and subsequently shared by several other media outlets and their associated social media profiles, leading to the snowball effect. In addition, the questionnaire was also shared among participants of the University of the Third Age in Poland. The inclusion criteria included Polish nationality and age ≥ 18 years. Specifically, the survey aimed to assess:

(i) familiarity of surveyed Poles with the concept of cultured meat;
(ii) willingness to purchase cultured meat if it were to be commercialized in Poland;
(iii) main reasons for acceptance or rejection of cultured meat;
(iv) potential concerns regarding cultured meat.

In order to ensure that all surveyed individuals understand the concept of cultured meat, including those who declared to be unfamiliar with it, the questionnaire displayed a plain language description of cultured meat accompanied by a graph showing the main steps of its production. In order to explore the characteristics that may potentially differentiate attitudes toward cultured meat, the questionnaire collected data on:

(i) factors influencing food purchases of surveyed individuals, including price, quality, taste, availability, origin, nutritional properties, animal welfare, and environmental impact;
(ii) patterns of meat and dairy consumption as assessed by declaration of dietary patterns;
(iii) awareness of adverse impacts of conventional meat production on the environment, including climate and quality of soil, water, and air;
(iv) openness to experience as assessed by the validated Polish adaptation of the Ten-Item Personality Inventory [38,39], as this personality trait is known to be associated with food choices [40,41]. Openness to experience was categorized into low and high based on normative data for the employed inventory [38];
(v) demographic characteristics, i.e., age, gender, level of education (none, primary, secondary, vocational, tertiary), place of residence (urban or rural area), and voivodeship (low and high GDP) [42].

The questionnaire was validated ad hoc and revised by qualified interdisciplinary experts. Given the size of the target population (population of Poles aged ≥ 18) [43], it was calculated using Cochran’s formula [44] that at least 1067 eligible individuals should be surveyed to reach a margin level of 3% at the confidence level of 95%. The research was approved by the Bioethics Committee at Poznan University of Medical Sciences (approval no. 828/21; date of approval: 4 November 2021).

### 2.2. Statistical Analyses and Calculations

The statistical analyses were performed using PQSTAT Software v.1.8.2 (PQStat Software, Poznan, Poland). Statistical analysis was performed on two groups: willing (the sum of decidedly yes and probably yes answers) and unwilling (the sum of decidedly no and probably no answers) to buy cultured meat. Fisher’s exact test was used to assess the willingness to purchase cultured meat in various subgroups. Variables found to affect willingness to purchase cultured meat in univariate analysis were selected for multiple logistic regression analysis. A *p*-value of less than 0.05 was considered statistically significant in all analyses.

## 3. Results

### 3.1. The Main Characteristics of the Studied Group

The demographic characteristics of the surveyed group are summarized in Table 1. Questionnaires obtained from 1553 adult Poles who completed a survey were considered in this study. The majority of those surveyed were female adults, were aged > 25 years, inhabited urban areas, and had tertiary education, which does not mirror the general population. By dietary patterns, omnivores were the most frequent group, while 21% of participants declared that they eliminate animal products to varying extents. Over half of the studied individuals scored low when tested for openness to experience (Table 1). The presented characteristics were further used in the analysis to understand their potential influence on cultured meat acceptance.

### 3.2. Factors Influencing Food Purchases

**Figure 1:**
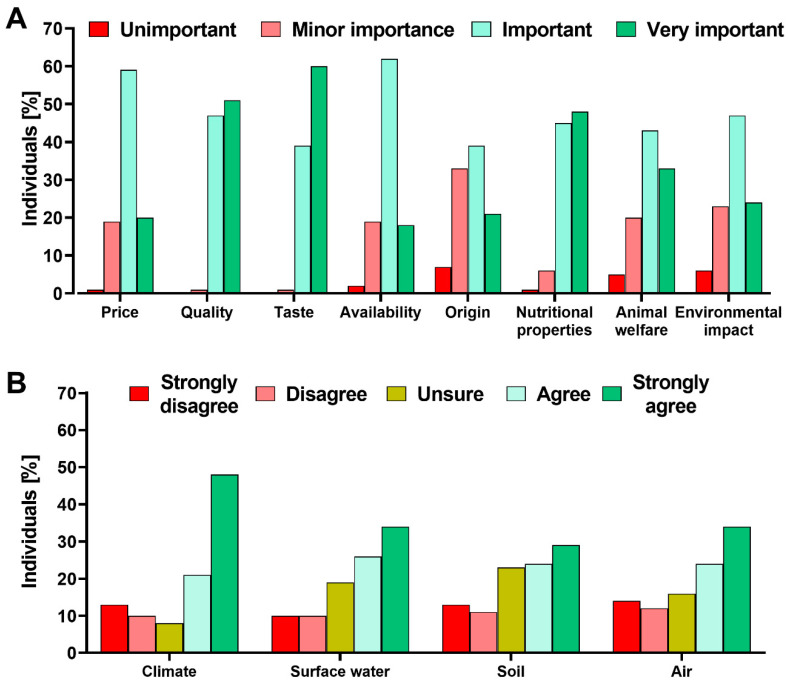
Opinion of studied individuals on (**A**) the importance of different factors during food purchase choices and (**B**) the opinion on adverse effects of animal farming on environmental compartments (*n* = 1553).

The majority (60%) of surveyed individuals indicated that the taste of food is a very important factor influencing their foodstuff purchases, followed by the quality of the product (51%) and nutritional properties (48%). Food availability and price were among the factors most frequently declared as important. The product’s origin, animal welfare, and environmental impact were most frequently considered unimportant factors during food purchases (Figure 1A).

### 3.3. Attitudes toward Environmental Impacts of Animal Farming

Most responders agreed that animal farming adversely affects the environment, with the majority indicating a negative impact on climate (69%), followed by water surface (60%), air (59%), and soil quality (53%). One-fourth of surveyed individuals (23–25%) disagreed that animal farming negatively affects air quality, soil, and climate. Moreover, one-fifth disagreed with the statement that animal farming negatively impacts surface waters (Figure 1B).

### 3.4. Cultured Meat Acceptance

Most respondents (63%) declared to be familiar with the concept of cultured meat definition and its production process, 26% had heard of cultured meat but lacked an understanding of how it is produced, and 11% had never heard of cultured meat. More than half (54%) of surveyed individuals were willing to purchase cultured meat products if commercialized, with 29% being definitive about it. However, over one-third declared no intention to buy such products, with approximately one-fifth of the surveyed being firm in this statement. Univariate analysis revealed that willingness to purchase cultured meat for consumption was more frequent among young adults (18–40 years), females, meat eaters, individuals with a high level of openness to experience, respondents previously familiar with the cultured meat concept, and those who agreed that animal farming adversely affects the climate and the quality of surface water, soil, and air (Table 2).

**Figure 2:**
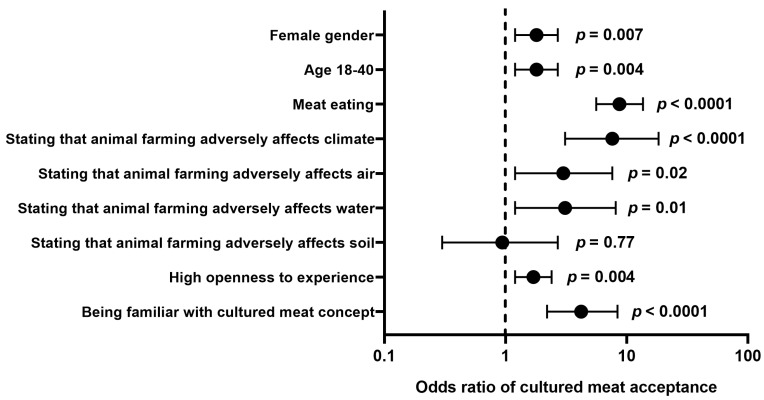
Logistic multiple regression results on the association between willingness to purchase cultured meat (presented as odds ratios with 95% confidence intervals) and characteristics of surveyed individuals selected based on the results of univariate analysis.

Multiple regression analysis further confirmed that being (i) a young adult (18–40 years), (ii) a woman, (iii) a meat eater, (iv) an individual with a high level of openness to experience, (v) familiar with the cultured meat concept, and (vi) an individual who agrees that animal farming has adverse effect on the climate and quality of surface waters and air were independent predictors of willingness to purchase cultured meat in the studied group (Figure 2).

**Figure 3:**
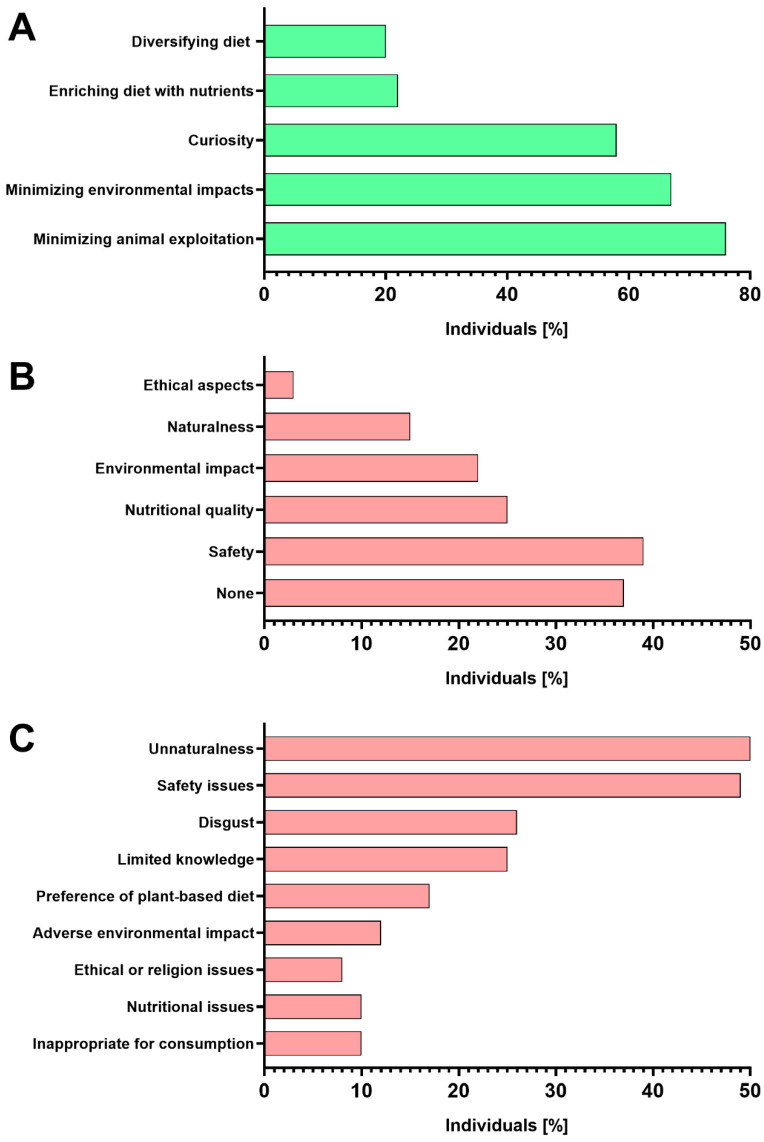
Motivation (**A**) and doubts (**B**) among respondents willing to purchase cultured meat (*n* = 846) and (**C**) reasons for cultured meat rejection among individuals unwilling to purchase it (*n* = 707).

In turn, the type and GDP of the inhabited area and education level did not affect willingness in univariate analysis and were therefore not included in the regression model. The primary motivations for buying cultured meat were related to minimizing the animal (76%) and environmental impacts of food production (67%). Moreover, over half of responders indicated to be driven by curiosity (Figure 3A). Over one-third of surveyed respondents declared no doubts or fears over cultured meat. Among those willing to purchase such meat, safety concerns (39%) were the most frequently declared fear, although over 20% also declared concerns over nutritional quality and environmental impacts (Figure 3B). The primary reasons for cultured meat rejection among those who were unwilling to purchase it in the future included its unnaturalness (50%) and concerns over the safety of its consumption (49%) (Figure 3C).

## 4. Discussion

The present study is the first to comprehensively assess the acceptance of cultured meat in the Polish population, providing essential data guiding food authorities and stakeholders that wish to introduce such products to the European market. The findings indicate that the Polish population may be moderately ready to accept the introduction of cultured meat products because over half of the studied individuals expressed the willingness to purchase them. Importantly, the results offer an overview of the main factors influencing the acceptance and rejection of cultured meat, which should be taken into account by stakeholders and food authorities when considering the authorization of such products for the market.

The essential finding of this study is that cultured meat is more likely to be accepted by those familiar with its concept, young adults, females, meat eaters, and individuals who are open to experience, as well as those informed on the environmental impacts of animal farming. Hence, they may constitute a primary target for such meat in Poland and, by the snowball effect of recommendations, influence the acceptance among family and friends [45,46]. Moreover, the acceptance of cultured meat may also become higher with increasing levels of public awareness of the environmental impacts of animal farming and the production of different types of meat, especially in terms of climate change, consumption of natural resources, and chemical pollution [47,48,49]. Other studies have already shown that environmental and ethical issues are one of the main drivers of willingness to choose cultured meat products [50,51,52,53].

Previous studies also indicated that younger people and females tend to buy or try to consume cultured meat [45,50]. Interestingly, various previous studies indicate that women may have lower acceptance of novel foods, such as those based on insects [54,55,56]. However, in this particular example, this effect may be due to a disgust sensitivity observed in women [57,58]. On the contrary, in the case of cultured meat, which shall also be regarded as a novel food, disgust may play a limited role, while higher levels of empathy evidenced in females [59] can be a driving force in accepting the solutions that decrease animal suffering. This hypothesis is supported by the higher pro-animal welfare attitudes compared to men, as evidenced in studies conducted across many countries [60,61,62,63,64].

The majority of respondents (89%) in the present study were familiar with the concept of cultured meat, which is in line with findings of studies conducted in other populations, e.g., Brazilian (73%) [50], French (72%) [52], Italian (66%) [65], and Chinese (70%) [45]. Contrary to this, research conducted in 2021 revealed that only 36% of surveyed inhabitants of South Africa were aware of cultured meat. The authors suggested that such low awareness may be due to insufficient media publicity and access to current information on innovative products [66]. The present study indicates that this parameter is related to increased willingness to purchase such meat. Similar observations were made previously in France, Germany, Italy, the Netherlands, and China [45,52,53,65,67]. This effect is likely related to the fear of novelty, which, from the evolutionary point of view, serves to avoid the dangers of possible injury or death while approaching a new resource, although its perception can be modified by increased information about it [68,69]. As demonstrated in the South African population, despite the initially low levels of familiarity with cultured meat, providing information about it during the questionnaire resulted in over half of the studied individuals expressing the willingness to consume it [66]. Therefore, spreading awareness about cultured meat technology should be regarded as an essential pathway to better acceptance [67].

Interestingly, though, the present study did not indicate that education level plays a role in the acceptance of cultured meat, contrary to previous surveys conducted, e.g., in China or South Africa [45,66,70]. One should note that better educational status is often a surrogate marker of improved access to evidence-based information in various populations. However, as shown in studies focusing on vaccine hesitancy, it is often not the only parameter explaining acceptance or rejection of technology [71]. It is suggested that a primary role in accepting novel products arising from scientific achievements may likely be played by honest, transparent, and balanced communication employing a language adjusted to particular demographical and occupational groups [72].

Transparent communication will be crucial regarding cultured meat, particularly if one considers that the present study clearly shows that safety issues are the primary concern, not only for individuals who do not accept cultured meat but also for those who are expressing a will to purchase it. Other consumer studies conducted in Australia, China, and Europe also highlighted that such meat’s safety is a primary concern [51,52,53,67,70]. Therefore, it is not only essential for food authorities to assess the safety of cultured meat, similarly to the premarketing evaluations conducted in 2022–2023 by the U.S. Food and Drug Administration for cultured poultry products [20,21], but they must also explicitly explain the basis of such assessment to the general public. In some populations, this may be a challenging task, e.g., in Poland, given the previous experience with genetically modified foods [25] or vaccines [37,73], which may emerge from relatively low trust in the government and public institutes [27,74]. Apart from safety concerns, an effort would be needed to address the apprehension over the unnaturalness of cultured meat, highlighted by individuals accepting and rejecting such meat. Consumers must realize that their perception of the naturalness of purchased food may be false and often arise from food marketing strategies [75].

In line with the majority of research conducted in Europe and China [45,52,65,70], the present study found that meat consumers were more frequently willing to buy or eat cultured meat than those who already exclude meat from their diet. This shows the high potential of cultured meat, the introduction of which to one’s menu does not require substantial changes and challenges related to switching to plant-based diets. Therefore, it may be a viable option for those who are unwilling to exclude meat consumption but wish to decrease the environmental impacts of its production and animal exploitation. One should note that this corresponds to the results of our study, which showed that these were the most frequent motivations to buy cultured meat. As shown, cultured meat can offer several environmental benefits, including significant reductions in water withdrawal, energy consumption, land use, and greenhouse emissions [76,77,78]. The recent analysis based on real-world data collected from over 15 companies and research institutes demonstrated that substantial benefits could be especially achieved when producing beef with a carbon footprint reduced by over 95% compared to the global average from conventional production in 2018 [18]. In the case of chicken production, the carbon footprint between conventional production and cultured meat was shown not to differ, but the latter was demonstrated to require less land surface and contribute less to acidification [18].

The present study highlights that Polish consumers perceive food’s taste, quality, and nutritional properties as important factors influencing their purchase choices. Therefore, to fulfill the consumers’ expectations, cultured meat must not only mimic the sensory properties of conventional counterparts but also match (or be superior to) the nutritional quality. One-fourth of individuals willing to purchase cultured meat in the present study indicated concerns over its nutritional value. Therefore, it is required to run comprehensive comparative studies, the results of which are also important to inform consumers using information on the label. According to the data presented to the U.S. Food and Drug Administration throughout the first premarketing assessment, compared to standard counterparts, serum-free cultured chicken revealed lower levels of total fat, saturated fat, monounsaturated and polyunsaturated fatty acids, and sodium; similar contents of moisture, total amino acids, niacin (B3), magnesium, and manganese; but higher levels of cholesterol, pantothenic acid (B5), pyridoxine (B6), vitamin A, calcium, iron, potassium, phosphorus, selenium, and zinc. It would be best to display these data on the product’s label for transparency, emphasizing the nutritional properties and ultimately building consumer trust [21,79]. There are also other aspects that may influence the acceptance of cultured meat, e.g., its shelf life, packaging, and how it is named in a particular language [79].

The present study also confirms openness to experience as an important personality trait influencing acceptance of novel foods such as cultured meat. Individuals with high openness may constitute a basis of knowledge spillovers [80] as they are more influenced by anchoring cues [81]. Therefore, the potential promotion campaigns could benefit from choosing a particularly important motivation in order to spread the high anchor, which will solidify support for cultured meat among people with high openness, which will constitute the foundation of the snowball effect. Importantly, over half of the individuals surveyed in the present study expressed curiosity as one of the drivers to purchase cultured meat. This has also been highlighted in previous studies exploring the acceptance of alternative protein sources, including insect-based and plant-based foods [70,82]. Curiosity, as an exploratory behavior, can also be a trigger to seek information related to novel foods, further increasing its acceptance by filling knowledge gaps [83]. However, curiosity alone, without any other motivations, e.g., ethical or environmental, may not be sufficient to convince individuals to purchase cultured meat regularly and cease the consumption of their conventional counterparts. Therefore, it might be beneficial for producers to put an emphasis on the sustainability and ethical performance of the offered products, ultimately maintaining the interest of the consumers [84,85].

Study limitations must be stressed. The research was based on an online survey, which allowed reaching out to a relatively large group of responders in a short time but did not allow for the verification of the responses on objective grounds. Moreover, it can be prone to volunteer bias, i.e., attract those interested in cultured meat more than those who reject this concept. Last but not least, declared (un)willingness to purchase the cultured meat expressed by surveyed individuals may not always reflect the actual decisions undertaken when such meat becomes available on the market as they can be affected by various factors, including the quality of the authorization process and communication of its outcomes to the general public.

## 5. Conclusions

The present study represents an overview of the perception of cultured meat products among Polish adults. It provides a guide for those interested in introducing such products to the market as it highlights the main factors influencing their acceptance (minimizing animal exploitation and environmental impacts) and rejection (safety and unnaturalness) as associated concerns that need to be addressed before, during, and after the authorization process. The results indicate that the Polish population may reveal moderate readiness for cultured meat because 54% of the surveyed individuals expressed willingness to buy, with only 29% being definitive about it. We argue that besides assessing food authorities, the commercialization of such meat requires balanced and transparent communication on its advantages and challenges. However, this will first require further studies evaluating cultured meat’s safety and nutritional quality and the impact of the production of its different types on various environmental compartments.

## Figures and Tables

**Table 1:** Main characteristics of the adult Polish population surveyed in this study (*n* = 1553).

General Characteristics	
**Gender**, Female/Male/Other, % (*n*)	78.3 (1217)/21.2 (329)/0.5 (7)
**Age**, mean (years ± SD)	38.3 ± 13.4
18–25 years, % (*n*)	15.6 (242)
26–30 years, % (*n*)	20 (310)
31–40 years, % (*n*)	30.5 (474)
41–50 years, % (*n*)	16.7 (259)
51–60 years, % (*n*)	7.8 (121)
>60 years, % (*n*)	9.5 (147)
**Place of residence**	
Urban area/Rural area, % (*n*)	85 (1314)/15 (239)
**Voivodeship Gross Domestic Product**	
Lower (<70 k)/Higher GDP (≥70 k), % (*n*)	52 (803)/48 (750)
**Education**	
None, % (*n*)	0.2 (3)
Primary, % (*n*)	0.6 (9)
Vocational, % (*n*)	1.7 (27)
Secondary, % (*n*)	22.9 (356)
Tertiary, % (*n*)	74.6 (1158)
**Diet type**	
Meat consumers, % (*n*)	78.9 (1225)
Omnivores, % (*n*)	49.4 (767)
Exclusion of dairy and eggs, % (*n*)	1.2 (19)
Flexitarianism, % (*n*)	28.3 (439)
Meat excluders, % (*n*)	21.1 (328)
Ovo-vegetarianism, % (*n*)	2.3 (36)
Lactoovovegetarianism, % (*n*)	12.7 (197)
Lactovegetarianism, % (*n*)	1.5 (23)
Veganism, % (*n*)	4.6 (72)
Personality trait	
Openness to Experiences, mean (SD)	5.1 (1.2)
High (≥5.38)/Low (<5.38), % (*n*)	48 (745)/52 (808)

**Table 2:** Association between surveyed characteristics and willingness to purchase cultured meat in the studied group (*n* = 1553). “Willing”/“Unwilling” categories include a sum of “probably yes/no” and “definitely yes/no”. The percentages may not add to 100% because unsure responders were excluded from the analysis.

Parameter	Subgroups	Willing	Unwilling	*p*-Value
		% (*n*)		
Age (years)	18–40	39.5 (613)	19.6 (304)	<0.00001
	>40	15.0 (233)	15.1 (235)	
Gender	Male	13.4 (208)	5.8 (92)	0.001
	Female	41 (636)	28.5 (443)	
Education	Non-tertiary	12.7 (197)	9.7 (151)	0.056
	Tertiary	41.5 (645)	24.9 (386)	
Inhabited area	Urban	46.3 (719)	29.2 (453)	0.647
	Rural	8.2 (127)	5.5 (86)	
Familiarity with the cultured meat concept	Yes	50.2 (779)	30.0 (462)	0.0027
	No	4.3 (66)	4.4 (68)	
Diet type	Meat eaters	45.1 (700)	25.8 (401)	0.0002
	Meat excluders	9.4 (146)	8.9 (138)	
Gross Domestic Product of inhabited region	Low (<70 k)	27.1 (421)	19.1 (296)	0.069
	High (≥70 k)	27.4 (425)	15.7(243)	
Attitudes toward conventional meat effects on the environment	Adversely affects climate	48.2 (749)	13.5 (210)	<0.00001
	Does not affect the climate	3.0 (47)	18.4 (286)	
	Adversely affects surface waters	40.7 (632)	13.1 (204)	<0.00001
	Does not affect surface waters	2.7 (42)	15.8 (245)	
	Affects soil quality	35.1 (545)	11.5 (178)	<0.00001
	Does not affect soil quality	4.8 (74)	17.3 (269)	
	Affects air quality	40.1 (623)	11.5 (178)	<0.00001
	Does not affect air quality	11.5 (178)	17.3 (269)	
Openness to experience	Low (<5.38)	25.5 (396)	20.0 (311)	0.0001
	High (≥5.38)	30.0 (450)	14.7 (228)	

## Data Availability

Data supporting reported results can be provided upon request from the corresponding author.

## References

[1] Nuñez I.A., Ross T.M. (2019). A Review of H5Nx Avian Influenza Viruses. Ther. Adv. Vaccines Immunother..

[2] Sánchez-Cordón P.J., Montoya M., Reis A.L., Dixon L.K. (2018). African Swine Fever: A Re-Emerging Viral Disease Threatening the Global Pig Industry. Vet. J..

[3] Fong I.W., Fong I.W. (2017). Animals and Mechanisms of Disease Transmission. Emerging Zoonoses: A Worldwide Perspective.

[4] Halabowski D., Rzymski P. (2021). Taking a Lesson from the COVID-19 Pandemic: Preventing the Future Outbreaks of Viral Zoonoses through a Multi-Faceted Approach. Sci. Total Environ..

[5] Mulchandani R., Wang Y., Gilbert M., Van Boeckel T.P. (2023). Global Trends in Antimicrobial Use in Food-Producing Animals: 2020 to 2030. PLoS Global Public Health.

[6] Murray C.J.L., Ikuta K.S., Sharara F., Swetschinski L., Aguilar G.R., Gray A., Han C., Bisignano C., Rao P., Wool E. (2022). Global Burden of Bacterial Antimicrobial Resistance in 2019: A Systematic Analysis. Lancet.

[7] Crippa M., Solazzo E., Guizzardi D., Monforti-Ferrario F., Tubiello F.N., Leip A. (2021). Food Systems Are Responsible for a Third of Global Anthropogenic GHG Emissions. Nat. Food.

[8] Machovina B., Feeley K.J., Ripple W.J. (2015). Biodiversity Conservation: The Key Is Reducing Meat Consumption. Sci. Total Environ..

[9] Poore J., Nemecek T. (2018). Reducing Food’s Environmental Impacts through Producers and Consumers. Science.

[10] Westhoek H., Lesschen J.P., Rood T., Wagner S., De Marco A., Murphy-Bokern D., Leip A., van Grinsven H., Sutton M.A., Oenema O. (2014). Food Choices, Health and Environment: Effects of Cutting Europe’s Meat and Dairy Intake. Glob. Environ. Chang..

[11] Alexander P., Brown C., Arneth A., Finnigan J., Rounsevell M.D.A. (2016). Human Appropriation of Land for Food: The Role of Diet. Glob. Environ. Chang..

[12] Our World in Data Yearly Number of Animals Slaughtered for Meat. https://ourworldindata.org/grapher/animals-slaughtered-for-meat.

[13] Michel F., Hartmann C., Siegrist M. (2021). Consumers’ Associations, Perceptions and Acceptance of Meat and Plant-Based Meat Alternatives. Food Qual. Prefer..

[14] van Huis A., Rumpold B. (2023). Strategies to Convince Consumers to Eat Insects? A Review. Food Qual. Prefer..

[15] Choi K.-H., Yoon J.W., Kim M., Lee H.J., Jeong J., Ryu M., Jo C., Lee C.-K. (2021). Muscle Stem Cell Isolation and in Vitro Culture for Meat Production: A Methodological Review. Compr. Rev. Food Sci. Food Saf..

[16] Hubalek S., Post M.J., Moutsatsou P. (2022). Towards Resource-Efficient and Cost-Efficient Cultured Meat. Curr. Opin. Food Sci..

[17] Rubio N.R., Xiang N., Kaplan D.L. (2020). Plant-Based and Cell-Based Approaches to Meat Production. Nat. Commun..

[18] Sinke P., Swartz E., Sanctorum H., van der Giesen C., Odegard I. (2023). Ex-Ante Life Cycle Assessment of Commercial-Scale Cultivated Meat Production in 2030. Int. J. Life Cycle Assess..

[19] SFA Growing Our Food Future. https://www.sfa.gov.sg/docs/default-source/publication/annual-report/sfa-ar-2020-20212c7b8b52e3e84fd193c56d53f42fe607.pdf.

[20] FDA (2023). FDA Completes Second Pre-Market Consultation for Human Food Made Using Animal Cell Culture Technology. https://www.fda.gov/food/cfsan-constituent-updates/fda-completes-second-pre-market-consultation-human-food-made-using-animal-cell-culture-technology.

[21] FDA (2023). FDA Completes First Pre-Market Consultation for Human Food Made Using Animal Cell Culture Technology. https://www.fda.gov/food/cfsan-constituent-updates/fda-completes-first-pre-market-consultation-human-food-made-using-animal-cell-culture-technology.

[22] USDA (2023). FSIS Directive 7800.1 FSIS Responsibilities in Establishments Producing Cell-Cultured Meat and Poultry Food Products. https://www.fsis.usda.gov/policy/fsis-directives/7800.1.

[23] EFSA The Safety of Cell Culture-Derived Food—Ready for Scientific Evaluation|EFSA. https://www.efsa.europa.eu/en/news/safety-cell-culture-derived-food-ready-scientific-evaluation.

[24] Cellular Agriculture Europe. https://www.cellularagriculture.eu/.

[25] Rzymski P., Królczyk A. (2016). Attitudes toward Genetically Modified Organisms in Poland: To GMO or Not to GMO?. Food Sec..

[26] Siegrist M., Hartmann C. (2020). Consumer Acceptance of Novel Food Technologies. Nat. Food.

[27] Sikora D., Rzymski P., Singh P., Borthakur A., Singh A.A., Kumar A., Singh K.K. (2021). Chapter 13—Public Acceptance of GM Foods: A Global Perspective (1999–2019). Policy Issues in Genetically Modified Crops.

[28] Slade P. (2018). If You Build It, Will They Eat It? Consumer Preferences for Plant-Based and Cultured Meat Burgers. Appetite.

[29] Siddiqui S.A., Alvi T., Sameen A., Khan S., Blinov A.V., Nagdalian A.A., Mehdizadeh M., Adli D.N., Onwezen M. (2022). Consumer Acceptance of Alternative Proteins: A Systematic Review of Current Alternative Protein Sources and Interventions Adapted to Increase Their Acceptability. Sustainability.

[30] Onwezen M.C., Bouwman E.P., Reinders M.J., Dagevos H. (2021). A Systematic Review on Consumer Acceptance of Alternative Proteins: Pulses, Algae, Insects, Plant-Based Meat Alternatives, and Cultured Meat. Appetite.

[31] Realini C.E., Ares G., Antúnez L., Brito G., Luzardo S., del Campo M., Saunders C., Farouk M.M., Montossi F.M. (2022). Meat Insights: Uruguayan Consumers’ Mental Associations and Motives Underlying Consumption Changes. Meat Sci..

[32] Siegrist M., Sütterlin B., Hartmann C. (2018). Perceived Naturalness and Evoked Disgust Influence Acceptance of Cultured Meat. Meat Sci..

[33] Krzysztoszek A. Poland Last EU Country to Make HPV Vaccine Free of Charge. https://www.euractiv.pl/section/zdrowie/news/poland-last-eu-country-to-make-hpv-vaccine-free-of-charge/.

[34] Nitsch-Osuch A., Gołębiak I., Wyszkowska D., Rosińska R., Kargul L., Szuba B., Tyszko P., Brydak L.B. (2017). Influenza Vaccination Coverage Among Polish Patients with Chronic Diseases. Adv. Exp. Med. Biol..

[35] Reczulska A., Tomaszewska A., Raciborski F. (2022). Level of Acceptance of Mandatory Vaccination and Legal Sanctions for Refusing Mandatory Vaccination of Children. Vaccines.

[36] Sobierajski T., Rzymski P., Wanke-Rytt M. (2023). Impact of the COVID-19 Pandemic on Attitudes toward Vaccination: Representative Study of Polish Society. Vaccines.

[37] Sowa P., Kiszkiel Ł., Laskowski P.P., Alimowski M., Szczerbiński Ł., Paniczko M., Moniuszko-Malinowska A., Kamiński K. (2021). COVID-19 Vaccine Hesitancy in Poland—Multifactorial Impact Trajectories. Vaccines.

[38] Gosling S.D., Rentfrow P.J., Swann W.B. (2003). A Very Brief Measure of the Big-Five Personality Domains. J. Res. Personal..

[39] Sorokowska A., Słowińska A., Zbieg A., Sorokowski P. (2014). Polska Adaptacja Testu Ten Item Personality Inventory (TIPI)—TIPI-PL—Wersja Standardowa i Internetowa. https://depot.ceon.pl/handle/123456789/5977.

[40] Ji M., Wong I.A., Eves A., Scarles C. (2016). Food-Related Personality Traits and the Moderating Role of Novelty-Seeking in Food Satisfaction and Travel Outcomes. Tour. Manag..

[41] Esposito C.M., Ceresa A., Buoli M. (2021). The Association Between Personality Traits and Dietary Choices: A Systematic Review. Adv. Nutr..

[42] GUS Rachunki Regionalne. https://stat.gov.pl/obszary-tematyczne/rachunki-narodowe/rachunki-regionalne/.

[43] GUS Ludność. Stan i Struktura Ludności Oraz Ruch Naturalny w Przekroju Terytorialnym. Stan w Dniu 31 Grudnia. https://stat.gov.pl/obszary-tematyczne/ludnosc/ludnosc/ludnosc-stan-i-struktura-ludnosci-oraz-ruch-naturalny-w-przekroju-terytorialnym-stan-w-dniu-31-grudnia,6,34.html.

[44] Cochran W. (1977). Sampling Techniques.

[45] Li H., Van Loo E.J., van Trijp H.C., Chen J., Bai J. (2023). Will Cultured Meat Be Served on Chinese Tables? A Study of Consumer Attitudes and Intentions about Cultured Meat in China. Meat Sci..

[46] Grasso A.C., Hung Y., Olthof M.R., Verbeke W., Brouwer I.A. (2019). Older Consumers’ Readiness to Accept Alternative, More Sustainable Protein Sources in the European Union. Nutrients.

[47] Lewisch L., Riefler P. (2023). Cultured Meat Acceptance for Global Food Security: A Systematic Literature Review and Future Research Directions. Agric. Food Econ..

[48] González N., Marquès M., Nadal M., Domingo J.L. (2020). Meat Consumption: Which Are the Current Global Risks? A Review of Recent (2010–2020) Evidences. Food Res. Int..

[49] Djekic I. (2015). Environmental Impact of Meat Industry—Current Status and Future Perspectives. Procedia Food Sci..

[50] Chriki S., Payet V., Pflanzer S.B., Ellies-Oury M.-P., Liu J., Hocquette É., Rezende-de-Souza J.H., Hocquette J.-F. (2021). Brazilian Consumers’ Attitudes towards So-Called “Cell-Based Meat”. Foods.

[51] Bogueva D., Marinova D. (2020). Cultured Meat and Australia’s Generation Z. Front. Nutr..

[52] Gousset C., Gregorio E., Marais B., Rusalen A., Chriki S., Hocquette J.F., Ellies-Oury M.P. (2022). Perception of Cultured “Meat” by French Consumers According to Their Diet. Livest. Sci..

[53] Bryant C., van Nek L., Rolland N.C.M. (2020). European Markets for Cultured Meat: A Comparison of Germany and France. Foods.

[54] Kröger T., Dupont J., Büsing L., Fiebelkorn F. (2022). Acceptance of Insect-Based Food Products in Western Societies: A Systematic Review. Front. Nutr..

[55] Ros-Baró M., Sánchez-Socarrás V., Santos-Pagès M., Bach-Faig A., Aguilar-Martínez A. (2022). Consumers’ Acceptability and Perception of Edible Insects as an Emerging Protein Source. Int. J. Environ. Res. Public Health.

[56] Andrić A., Miličić M., Bojanić M., Obradović V., Šašić Zorić L., Petrović M., Gadjanski I. (2023). Survey on Public Acceptance of Insects as Novel Food in a Non-EU Country: A Case Study of Serbia. J. Insects Food Feed.

[57] Egolf A., Siegrist M., Hartmann C. (2018). How People’s Food Disgust Sensitivity Shapes Their Eating and Food Behaviour. Appetite.

[58] Hartmann C., Siegrist M. (2016). Becoming an Insectivore: Results of an Experiment. Food Qual. Prefer..

[59] Greenberg D.M., Warrier V., Abu-Akel A., Allison C., Gajos K.Z., Reinecke K., Rentfrow P.J., Radecki M.A., Baron-Cohen S. (2023). Sex and Age Differences in “Theory of Mind” across 57 Countries Using the English Version of the “Reading the Mind in the Eyes” Test. Proc. Natl. Acad. Sci. USA.

[60] Graça J., Calheiros M.M., Oliveira A., Milfont T.L. (2018). Why Are Women Less Likely to Support Animal Exploitation than Men? The Mediating Roles of Social Dominance Orientation and Empathy. Personal. Individ. Differ..

[61] Apostol L., Rebega O.L., Miclea M. (2013). Psychological and Socio-Demographic Predictors of Attitudes toward Animals. Procedia—Soc. Behav. Sci..

[62] Herzog H.A. (2007). Gender Differences in Human-Animal Interactions: A Review. Anthrozoös.

[63] Clark B., Stewart G.B., Panzone L.A., Kyriazakis I., Frewer L.J. (2016). A Systematic Review of Public Attitudes, Perceptions and Behaviours Towards Production Diseases Associated with Farm Animal Welfare. J. Agric. Env. Ethics.

[64] Randler C., Adan A., Antofie M.-M., Arrona-Palacios A., Candido M., Boeve-de Pauw J., Chandrakar P., Demirhan E., Detsis V., Di Milia L. (2021). Animal Welfare Attitudes: Effects of Gender and Diet in University Samples from 22 Countries. Animals.

[65] Mancini M.C., Antonioli F. (2019). Exploring Consumers’ Attitude towards Cultured Meat in Italy. Meat Sci..

[66] Falowo B.A., Hosu Y.S., Idamokoro E.M. (2022). Perspectives of Meat Eaters on the Consumption of Cultured Beef (in Vitro Production) From the Eastern Cape of South Africa. Front. Sustain. Food Syst..

[67] Rolland N.C.M., Markus C.R., Post M.J. (2020). The Effect of Information Content on Acceptance of Cultured Meat in a Tasting Context. PLoS ONE.

[68] Langfield T., Shackelford T.K., Weekes-Shackelford V.A. (2021). Neophobia. Encyclopedia of Evolutionary Psychological Science.

[69] Resh M.D. (2013). Covalent Lipid Modifications of Proteins. Curr. Biol..

[70] Liu J., Hocquette É., Ellies-Oury M.-P., Chriki S., Hocquette J.-F. (2021). Chinese Consumers’ Attitudes and Potential Acceptance toward Artificial Meat. Foods.

[71] Rzymski P., Poniedziałek B., Fal A. (2021). Willingness to Receive the Booster COVID-19 Vaccine Dose in Poland. Vaccines.

[72] Rzymski P., Borkowski L., Drąg M., Flisiak R., Jemielity J., Krajewski J., Mastalerz-Migas A., Matyja A., Pyrć K., Simon K. (2021). The Strategies to Support the COVID-19 Vaccination with Evidence-Based Communication and Tackling Misinformation. Vaccines.

[73] Sobierajski T., Rzymski P., Wanke-Rytt M. (2023). The Influence of Recommendation of Medical and Non-Medical Authorities on the Decision to Vaccinate against Influenza from a Social Vaccinology Perspective: Cross-Sectional, Representative Study of Polish Society. Vaccines.

[74] Poland: Trust in Public Institutions 2020. https://www.statista.com/statistics/1193389/poland-trust-in-public-institutions/.

[75] Michel F., Sanchez-Siles L.M., Siegrist M. (2021). Predicting How Consumers Perceive the Naturalness of Snacks: The Usefulness of a Simple Index. Food Qual. Prefer..

[76] Tuomisto H.L., Allan S.J., Ellis M.J. (2022). Prospective Life Cycle Assessment of a Bioprocess Design for Cultured Meat Production in Hollow Fiber Bioreactors. Sci. Total Environ..

[77] Tuomisto H.L., Teixeira de Mattos M.J. (2011). Environmental Impacts of Cultured Meat Production. Environ. Sci. Technol..

[78] Lynch J., Pierrehumbert R. (2019). Climate Impacts of Cultured Meat and Beef Cattle. Front. Sustain. Food Syst..

[79] Siddiqui S.A., Bahmid N.A., Karim I., Mehany T., Gvozdenko A.A., Blinov A.V., Nagdalian A.A., Arsyad M., Lorenzo J.M. (2022). Cultured Meat: Processing, Packaging, Shelf Life, and Consumer Acceptance. LWT.

[80] Obschonka M., Tavassoli S., Rentfrow P.J., Potter J., Gosling S.D. (2023). Innovation and Inter-City Knowledge Spillovers: Social, Geographical, and Technological Connectedness and Psychological Openness. Res. Policy.

[81] McElroy T., Dowd K. (2007). Susceptibility to anchoring effects: How openness-to-experience influences responses to anchoring cues. Judgm. Decis. Mak..

[82] Estell M., Hughes J., Grafenauer S. (2021). Plant Protein and Plant-Based Meat Alternatives: Consumer and Nutrition Professional Attitudes and Perceptions. Sustainability.

[83] Piochi M., Micheloni M., Torri L. (2022). Effect of Informative Claims on the Attitude of Italian Consumers towards Cultured Meat and Relationship among Variables Used in an Explicit Approach. Food Res. Int..

[84] Lin-Hi N., Reimer M., Schäfer K., Böttcher J. (2023). Consumer Acceptance of Cultured Meat: An Empirical Analysis of the Role of Organizational Factors. J. Bus. Econ..

[85] Siddiqui S.A., Khan S., Murid M., Asif Z., Oboturova N.P., Nagdalian A.A., Blinov A.V., Ibrahim S.A., Jafari S.M. (2022). Marketing Strategies for Cultured Meat: A Review. Appl. Sci..

